# Normal value of neuron-specific enolase for predicting good neurological outcomes in comatose out-of-hospital cardiac arrest survivors

**DOI:** 10.1371/journal.pone.0305771

**Published:** 2024-06-25

**Authors:** Dongju Kim, Hyojeong Kwon, Sang-Min Kim, June-Sung Kim, Youn-Jung Kim, Won Young Kim

**Affiliations:** Department of Emergency Medicine, Asan Medical Center, University of Ulsan College of Medicine, Seoul, Republic of Korea; CHU Nantes, FRANCE

## Abstract

Research on prognostic factors for good outcomes in out-of-hospital cardiac arrest (OHCA) survivors is lacking. We assessed whether normal levels of normal neuron-specific enolase (NSE) value would be useful for predicting good neurological outcomes in comatose OHCA survivors treated with targeted temperature management (TTM). This registry-based observational study with consecutive adult (≥18 years) OHCA survivors with TTM who underwent NSE measurement 48 hours after cardiac arrest was conducted from October 2015 to November 2022. Normal NSE values defined as the upper limit of the normal range by the manufacturer (NSE <16.3 μg/L) and guideline-suggested (NSE < 60 μg/L) were examined for good neurologic outcomes, defined as Cerebral Performance Categories ≤2, at 6 months post-survival. Among 226 OHCA survivors with TTM, 200 patients who underwent NSE measurement were enrolled. The manufacturer-suggested normal NSE values (<16.3 μg/L) had a specificity of 99.17% for good neurological outcomes with a very low sensitivity of 12.66%. NSE <60 μg/L predicted good outcomes with a sensitivity of 87.34% and specificity of 72.73%. However, excluding 14 poor-outcome patients who died from multi-organ dysfunction excluding hypoxic brain injury, the sensitivity and specificity of normal NSE values were 12.66% and 99.07% of NSE < 16.3 μg/L, and 87.34% and 82.24% of NSE < 60 μg/L. The manufacturer-suggested normal NSE had high specificity with low sensitivity, but the guideline-suggested normal NSE value had a comparatively low specificity for good outcome prediction in OHCA survivors. Our data demonstrate normal NSE levels can be useful as a tool for multimodal appropriation of good outcome prediction.

## Introduction

Outcome prediction after cardiac arrest is crucial in aiding the decision to continue with or withdraw from intensive care [1]. Numerous prognostic studies in out-of-hospital cardiac arrest (OHCA) survivors have focused on unfavorable outcome prediction to avoid pursuing ineffective treatment. Presently, the prognostic algorithms recommended by current guidelines suggest a high likelihood of unfavorable outcomes (false positive rate < 5%) [2, 3]. However, most OHCA survivors do not fulfill the guideline criteria for a poor outcome, leaving them with an intermediate prognosis [4–8]. Predicting favorable outcomes is extremely challenging. However, a good prognosis can reduce uncertainty, help inform decisions about the escalation of organ support [9, 10], and counterbalance a falsely pessimistic signal from predictors of poor neurological outcomes [9, 11].

Serum neuron-specific enolase (NSE), which reflects neuronal cell damage and hypoxic-ischemic brain injury, is the only recommended biomarker [3]. Current guidelines suggest an NSE threshold of > 60 μg/L at 48 or 72 hours for predicting poor outcomes in a multimodal prognostication algorithm [1, 2]. However, research on the normal NSE value for good neurological outcomes in OHCA survivors is lacking.

This study therefore aimed to assess the prognostic ability of the normal NSE value defined as the upper limit of normal (NSE < 16.3 μg/L) or with a guideline-suggested cut-off value (< 60 μg/L) to predict good neurological outcomes in OHCA survivors.

## Materials and methods

### Study design and population

This retrospective cohort study using prospectively collected TTM registry data was performed at an urban academic adult emergency department at a tertiary referral center in South Korea from October 2015 to November 2022. The inclusion criteria of the TTM registry were comatose adult (aged > 18 years) patients with non-traumatic OHCA who were treated with TTM, and the exclusion criteria were patients with active intracranial bleeding or acute ischemic stroke, limitations in therapy, and a do-not-attempt resuscitation order, cerebral performance category (CPC) 3 or 4 before OHCA, and unknown outcomes for 6 months after the return of spontaneous circulation (ROSC). Among the eligible patients in the TTM registry, this study included those who underwent serum NSE level 48 hours after cardiac arrest. All the enrolled patients received post-resuscitation care per the current Advanced Cardiac Life Support guidelines. The Institutional Review Board of Asan Medical Center reviewed and approved the study protocol, and informed consent was waivered due to the use of collected registry data (IRB No-2023-0769).

### Data collection and outcomes

Demographic and clinical data were extracted from the web-based registry. The variables investigated in this study were: age, sex, medical history, witnessed arrest, arrest cause, initial documented rhythm, time from collapse to ROSC, initial vital signs, and outcome. Laboratory values at admission were retrieved from the TTM registry [8]. The researchers conducted clinical follow-ups to determine the neurologic status according to the CPC score at discharge and 6 months after cardiac arrest through face-to-face visits or standardized follow-up telephone interviews. Manufacturer-suggested normal NSE level was defined as the upper limit of the normal range (NSE < 16.3 μg/L), and the guideline-suggested normal NSE level was defined as NSE < 60 μg/L. The primary outcome was good neurological outcome at 6 months after cardiac arrest, defined as a CPC score of 1 or 2. Furthermore, a poor neurological outcome was designated by a CPC score ranging from 3 to 5.

### Statistical analyses

Continuous variables are presented as means ± standard deviation (normal distribution) or median with interquartile range (abnormal distribution). The normal distribution of continuous variables was assessed using the Kolmogorov–Smirnov test. Categorical variables are expressed as numbers and percentages. To analyze the baseline characteristics and laboratory examinations between the poor and good neurological outcome groups, Student’s *t*-test was used to compare the means of normally distributed continuous variables, and the Mann–Whitney U-test was used to compare the non-normally distributed continuous variables. The Chi-squared or Fisher’s exact test was used to compare categorical variables. The 95% confidence intervals (CIs) were examined to determine the performance of the NSE level at 48 hours after cardiac arrest in predicting a good neurological outcome at 6 months. We evaluated the prognostic value of normal NSE levels for good neurologic outcomes at 6 months by the sensitivity, specificity, positive, and negative predictive values. To evaluate the relationship between NSE level groups (< 16.3 μg/L, ≥ 60 μg/L, and values in between) and good neurological outcome, we performed Cox regression analysis, calculating hazard ratios (HR) and their 95% confidence intervals (CIs) for good neurological outcome. We included variables that were significant in the univariate analysis (p-value < 0.1) in the multivariate model after confirming the absence of multicollinearity through linear regression analysis, as indicated by VIF values less than 2.5. A two-tailed p-value of <0.05 was considered indicative of statistical significance. Statistical analyses were performed using SPSS for Windows, version 20.0 (SPSS Inc., Chicago, IL, USA).

## Results

**Fig 1:**
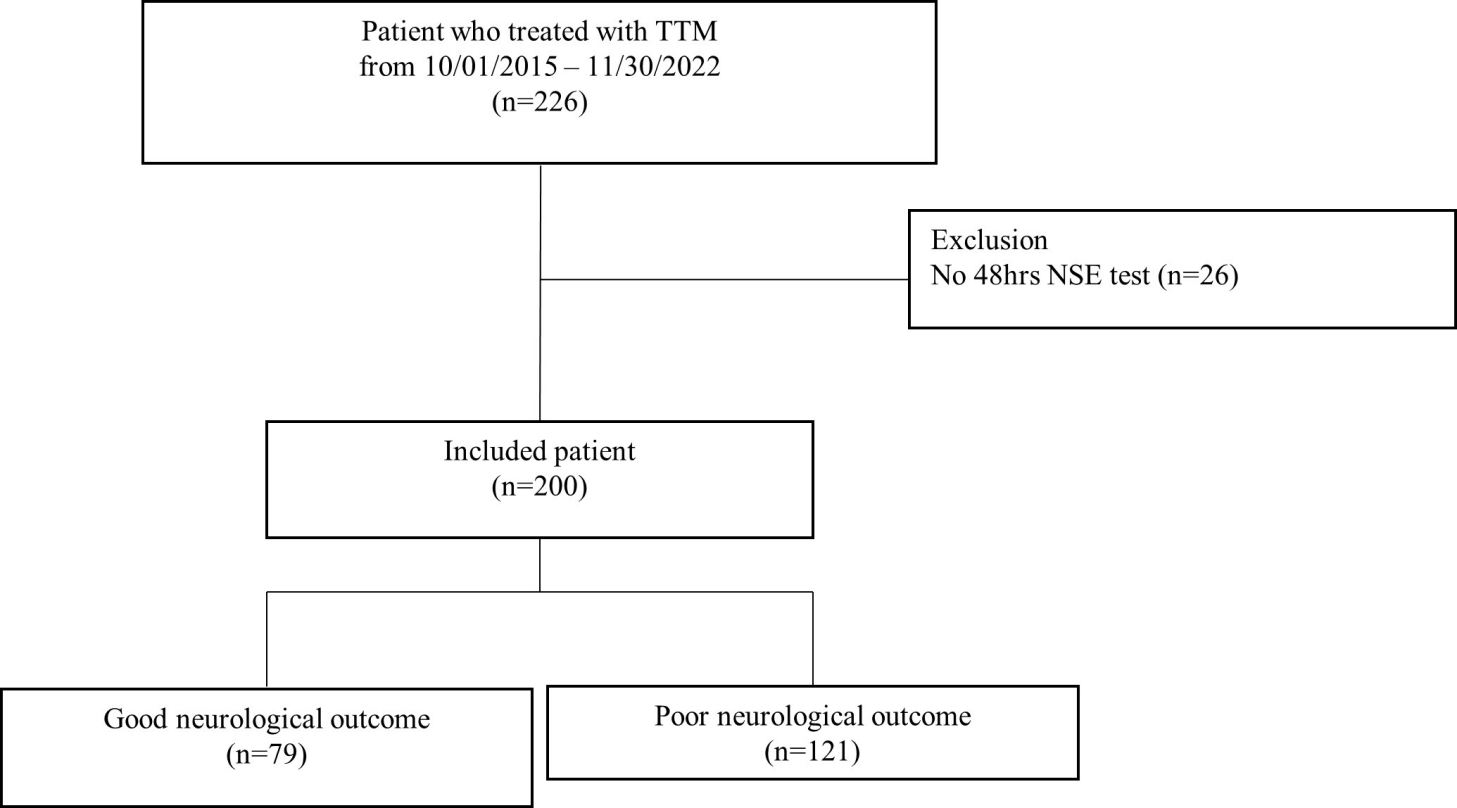
Study flow chart. Abbreviations: TTM, Targeted temperature management; NSE, Neuron-specific enolase.

Of the 226 consecutive patients treated with TTM after OHCA, we excluded 26 who did not have measurements of serum NSE levels at 48 hours after cardiac arrest. Finally, 200 patients were included. Good neurological outcomes were observed in 79 (39.5%) patients (Fig 1). Their mean age was 60.6± 15.3 years, and 142 (71%) were male.

Compared with the good outcomes group, the poor neurological outcome group was older (63.5±14.6 vs. 56.1±15.2 years, p<0.001), and had a lower occurrence of witnessed cardiac arrest (74.3% vs. 89.9%, p = 0.007), presumed cardiac cause (47.1% vs. 87.3%, p<0.001), and shockable initial rhythms (22.3% vs. 68.4%, p<0.001) (Table 1).

**Table 1:** Baseline demographics and clinical characteristics between the poor and good neurological outcome groups.

	Total (n = 200)	Good neurological outcome (n = 79)	Poor neurological outcome (n = 121)	P value
Age	60.6±15.3	56.1±15.2	63.5±14.6	<0.001
Sex (male)	142 (71%)	58 (73.4%)	84 (69.4%)	0.543
Witnessed arrest	161(80.5%)	71 (89.9%)	90 (74.3%)	0.007
Bystander CPR	142 (71%)	56 (70.9%)	86 (71.1%)	0.977
**Prehospital initial rhythm**				<0.001
Shockable	81 (40.5%)	54 (68.4%)	27 (22.3%)	
Non-shockable	107 (53.5%)	19 (24.1%)	88 (72.7%)	
Unknown	12 (6%)	6 (7.6%)	6 (4.9%)	
**Arrest cause**				<0.001
Presumed cardiac cause	126 (63%)	69 (87.3%)	57 (47.1%)	
Other medical cause	43 (21.5%)	5 (6.3%)	38 (31.4%)	
Asphyxia	14 (7%)	3 (3.8%)	11 (9.1%)	
Hanging	11 (5.5%)	2 (2.5%)	9 (7.4%)	
Other cause	6 (3%)	0 (0%)	6 (4.9%)	
**Previous medical history**				
Hypertension	82 (41%)	30 (38.0%)	52 (43.0%)	0.482
DM	51(25.5%)	15(19.0%)	36 (29.8%)	0.088
Arrhythmia	22 (11%)	11 (13.9%)	11 (9.1%)	0.286
Acute coronary syndrome	13 (6.5%)	5 (6.3%)	8 (6.6%)	0.925
**Vital signs**				
Systolic pressure, mmHg	143.1±50.6	143.4±47.4	142.1±52.3	0.856
Diastolic pressure, mmHg	80.0±28.5	84.0±25.9	76.9±29.7	0.096
Pulse rate, beats/min	102.9±32.7	100.9±29.5	104.5±34.9	0.472
**Laboratory findings, initial**				
White blood cell, **10^3^**/μL	12.6 [9.7–16.5]	12.5[10.2–15.9]	12.8[8.7–16.8]	0.741
Hemoglobin, g/dL	12.9 [10.8–14.5]	13.9 [12.2–15.2]	12.1 [10.2–14.0]	<0.001
Platelet, **10^3^**/μL	203.0 [146.2–252.7]	213.0 [170.0–278.0]	191.0 [140.0–240.0]	0.027
BUN, mg/mL	18 [14.0–26.0]	17.0 [14.0–22.0]	19.0 [14.5–29.0]	0.022
Creatinine, mg/dL	1.25 [1.00–1.67]	1.19 [1.03–1.45]	1.29 [0.99–1.95]	0.101
AST, IU/L	126.0 [73.3–227.3]	119.0 [76.0–186.0]	138.0 [71.5–240.5]	0.331
ALT, IU/L	85.5 [41.5–161.8]	80.0 [49.0–141.0]	99.0 [37.0–178.5]	0.708
Total bilirubin, mg/dL	0.4 [0.3–0.6]	0.5 [0.3–0.7]	0.4 [0.2–0.6]	0.005
ECMO	28 (14.0%)	15 (19.0%)	13 (10.7%)	0.100
NSE at 48 hours, μg/L	56.6 [28.0–278]	27.6 [19.1–46.4]	215.9 [54.8–485.3]	0.001

All values are presented as the mean±SD, median[interquartile rage] or as numbers (percentages).

Abbreviations: CPR, cardiopulmonary resuscitation; DM, diabetes mellitus; BUN, blood urea nitrogen; AST, aspartate aminotransferase; ALT, alanine aminotransferase; ECMO, extracorporeal membrane oxygenation.

The manufacturer-suggested normal NSE value (<16.3μg/L) could predict good neurological outcomes in 11 of 200 patients, and 189 of 200 for poor neurological outcomes. The sensitivity, specificity, and positive and negative predictive values of the manufacturer-suggested normal NSE value were 12.7%(95% CI; 6.2–22.1), 99.2%(95% CI; 95.5–100.0), 90.9%(95% CI; 56.6–98.7), and 63.5%(95% CI; 61.5–65.5), respectively. However, NSE <60μg/L identified 102 patients for good outcomes and 98 for poor outcomes. The sensitivity, specificity, and positive and negative predictive values were 87.3%(78.0–93.8), 72.7%(63.9–80.4), 67.7%(60.7–73.9), and 89.8%(83.0–94.1), respectively (Table 2).

**Table 2:** Prognostic ability of normal neuron-specific enolase (NSE) levels for good neurological outcomes.

NSE at 48 hours	Accuracy (95% CI)	Sens (95% CI)	Spec (95% CI)	PPV (95% CI)	NPV (95% CI)	Patients, n			
						TP	FP	TN	FN
**NSE <16.3 μg/L**	65.0 (58.0–71.6)	12.7 (6.2–22.1)	99.2 (95.5–100.0)	90.9 (56.6–98.7)	63.5 (61.5–65.5)	10	1	120	69
**NSE**≤41** μg/L**	78.5 (72.3–83.6)	73.4 (62.8–81.9)	81.8 (74.0–87.7)	72.5 (61.9–81.1)	82.5 (74.7–88.3)	58	22	99	21
**NSE <60 μg/L**	78.5 (72.2–84.0)	87.3 (78.0–93.8)	72.7 (63.9–80.4)	67.7 (60.7–73.9)	89.8 (83.0–94.1)	69	33	88	10

All values are presented as percentages or numbers.

Abbreviations: NSE, neuron-specific enolase; AUC, area under curve; Sens, sensitivity; Spec, specificity; PPV, positive predictive value; NPV, negative predictive value; TP, true positive; FP, false positive; TN, true negative; FN, false negative; CI, confidential interval.

Among the 31 patients with poor outcomes with NSE <60 μg/L, 14 (41.9%) died from other organ injuries excluding hypoxic brain injury. Of these, eight had poor neurological outcomes due to complications of extracorporeal treatment. Specifically, two patients who received ECMO developed heparin-induced thrombocytopenia (HIT), which led to life-threatening bleeding. Five patients were not weaned from the ventilator due to ventilator-associated pneumonia, and one patient who received CRRT developed HIT, which led to intraventricular hemorrhage. Four of the 14 patients died from underlying disease, including septic shock and uncontrolled gastrointestinal bleeding. Two patients had poor outcomes due to unexpected conditions, including osmotic demyelination syndrome and in-hospital cardiac arrest during intra-hospital transport.

Excluding 14 poor-outcome patients who died not from hypoxic brain injury but multi-organ dysfunction, the sensitivity and specificity of manufacturer-suggested normal NSE values did not change, but the specificity of guideline-suggested normal NSE value increased from 72.7% to 82.2%.

In the univariate analysis, unadjusted hazard ratios (HR) for good neurological outcome were 23.57 (95% CIs, 9.41–59.03) for the NSE<16.3 μg/L group and 9.70 (95% CIs, 4.95–18.99) for the intermediate values group. After adjusting for statistically significant covariates from the univariate analysis, the HR for good neurological outcome remained significantly higher in the NSE<16.3 μg/L group (adjusted HR, 21.33; 95% CIs, 8.20–55.51) (Table 3).

**Table 3:** Unadjusted and adjusted hazard ratio and 95% confidence intervals (95% CIs) for good neurological outcome.

	Crude model		Adjusted model	
	HR (95% CIs)	P-value	HR (95% CIs)	P-value
**NSE(≥60 μg/L)**	Reference		Reference	
**NSE(16.3 μg/L ≥, <60 μg/L)**	9.70 (4.95–18.99)	<0.001	6.53 (3.24–13.16)	<0.001
**NSE(<16.3 μg/L)**	23.57 (9.41–59.03)	<0.001	21.33 (8.20–55.51)	<0.001
Age	0.97 (0.96–0.99)	<0.001	0.98 (0.96–0.99)	0.002
Witnessed arrest	2.48 (1.19–5.15)	0.015	1.06 (0.49–2.29)	0.888
Initial shockable rhythm	4.46 (2.77–7.18)	<0.001	2.09 (1.15–3.79)	0.015
Presumed cardiac cause	5.20 (2.68–10.11)	<0.001	2.21 (0.98–4.98)	0.054
Platelets at admission	1.00 (1.00–1.01)	0.019	1.00 (1.00–1.01)	0.291
**Likelihood Ratio test: 119.3, P-value<0.001**				

Adjusted model: adjusted for age, witnessed cardiac arrest, initial shockable rhythm, Presumed cardiac cause, platelets at admission

Abbreviations: HR, hazard ratio; CIs, confidence interval; ROSC, return of spontaneous circulation.

## Discussion

This study aimed to assess the prognostic ability of normal NSE value defined according to manufacturer-suggested (NSE <16.3 μg/L) or guideline-suggested (<60 μg/L) cut-off values to predict good neurological outcomes in OHCA. We found that the manufacturer-suggested normal NSE values had a high specificity but unacceptably low sensitivity of 12.7% for predicting good neurological outcomes. Therefore, patients with NSE <16.3 μg/L will likely have favorable neurological outcomes with a positive predictive value of 90.9%. The guideline-suggested normal NSE value had high sensitivity (87.3%) with a negative predictive value of 89.8%, while the specificity of 72.7% was insufficient. However, excluding 14 patients with poor outcomes who died from multi-organ dysfunction, the specificity of the guideline-suggested normal NSE value increased to 82.2%.

Current international guidelines recommend a multimodal approach and delayed timing (72hours after cardiac arrest) of prognostication to minimize the possibility of inappropriate withdrawal of life-sustaining therapy (WLST) for patients who may otherwise achieve meaningful neurological recovery. An NSE threshold level of > 60 μg/L at 48 or 72 hours is also included in the multimodal prognostication algorithm for predicting poor outcomes [2]. Despite the recommended guidelines, early prediction of good neurologic outcomes may offer information for caregivers and physicians, guiding discussions on invasive care plans. such as extracorporeal membrane oxygenation (ECMO) or emergency coronary artery bypass graft (CABG) in comatose OHCA survivors [7, 11–13].

Recent systematic review cautiously mention low NSE or NFL blood values and normal MRI suggesting a potential good outcome [9]. Biomarkers, including NSE, glial protein S-100B, neurofilament light chain (NFL), glial fibrillary acidic protein (GFAP), tau protein, and ubiquitin carboxy-terminal hydrolase-L1 (UCH-L1), have been examined for their prognostic value. S-100B exhibits lower specificity compared to NSE, along with low sensitivity [11]. NFL and GFAP, however, demonstrate both high specificity and comparatively higher sensitivity than other biomarkers [11, 14]. Nonetheless, their limited availability presents a challenge for widespread use [15].

NSE remains the most extensively studied biomarker in neuro-prognostication following cardiac arrest. Previous research has identified normal NSE levels as 16–17 μg/L, using this range as a cutoff to predict good neurological outcomes at six months with over 80% specificity, even when measured just 24 hours after ROSC [11, 16]. However, the determination of optimal timing and consistent threshold values for NSE is still unresolved.

In this study, we found that the guideline-suggested NSE cut-off value to predict good neurological outcomes has a high sensitivity (87.3%) and negative predictive value of 89.8%, suggesting false negatives in 10 patients, which may be useful to screen for the decision of resource-intensive inclusion care. This is supported by a recent study examining the usefulness of novel serum biomarkers in OHCA survivors with TTM [17]. They reported normal NSE at 48 hours had a sensitivity of 90.2%, specificity of 35.6%, and negative predictive value of 80.0%. However, we found that the manufacturer-suggested normal NSE value correctly predicted good outcomes with a specificity of 99.2% and a positive predictive value of 90.9%. These patients will likely recover from brain damage and require continuous treatment without WLST. Our results are similar to those of Streitberger et al. [18], who reported that NSE ≤ 17 μg/L at 72 hours had a negative predictive value for CPC 4–5 of 92%, and sensitivity for the detection of CPC 1–3 was 33%.

Although the specificity of guideline-suggested NSE cut-off value for good outcomes was lacking (72.7%) in our findings, when excluding patients who died from causes excluding hypoxic brain injury (14/31), specificity increased to 82.3%. This is consistent with the report that in five of the 14 patients with CPC 4–5, despite NSE ≤ 17 μg/L, clinical findings proved an absence of severe hypoxic-ischemic encephalopathy [18].

The most recent study indicated that the highest NSE value within 48 hours, if ≤41μg/L, demonstrated a sensitivity of 91.48% and a specificity of 58.36% [19]. In contrast, our results showed comparatively lower sensitivity but higher specificity. We adhered to a strict 48-hour timeframe for measuring NSE levels, whereas the referenced study considered peak NSE values. Additionally, in our cohort, the median NSE level among those with poor neurological outcomes was higher compared to their cohort. This difference may account for the observed discordance in sensitivity and specificity between the two studies.

Our study has several limitations. First, our results were based on a retrospective cohort, involving a relatively small patient group treated at a single tertiary hospital. Consequently, the generalization of our findings to other institutions or patient populations is limited. Second, we measured NSE at a single time point rather than using serial measurements. NSE is a biomarker that exhibits dynamic changes over time, so serial measurements may provide a more accurate assessment of neurological outcomes. Third, our study did not account for the potential hemolytic effects, including those associated with ECMO. Our center uses the hemolysis index when reporting NSE values; however, this could potentially confound our findings. Lastly, the usefulness of serum NSE value is assessed by multimodal approaches, including other tests; however, this was not the case in our study. Further study in a larger patient cohort is warranted.

## Conclusion

We found that manufacturer-suggested normal NSE had a high specificity with low sensitivity, but guideline-suggested normal NSE value had a comparatively low specificity for good outcome prediction in OHCA survivors. Two normal NSE levels can be useful as a tool, appropriately depending on the need for the sensitivity and specificity, for multimodal appropriation of good outcome prediction.
